# Contract Medical Practice

**Published:** 1902-10-11

**Authors:** 


### Hospital Clinics and Medical Progress.

### CONTRACT MEDICAL PRACTICE.

Among the various groups of people who, from within and from without, wish to reform the medical profession and to alter if not improve the relations between the doctors and the public, a strong feeling of antagonism seems to have grown up against what is called "contract medical service" in every form. Theoretically and as a counsel of perfection we agree with them. Obviously the right and proper thing in a well-ordered world would be that each patient should come with his fee in his hand, and should offer it without question Unfortunately the poor, who do not as a rule possess banking accounts, are always with us, and they experience much difficulty in finding the fee just when it is wanted; and this is the cause of many complications, many bad debts, and much hospital abuse. One way out of the difficulty, and one which is peculiarly adapted to the requirements of the weekly wage-earning classes, is the so-called "provident or penny-a-week system; a system according to which in return for a fixed payment, ill or well, the doctor undertakes to attend when illness comes. And it is against all such contracts or undertakings that certain members of the British Medical Association have lately raised a protest and passed a resolution. Yet what are poor people to do? We must take them as they are, with their habits, prejudices, and customs; and one of their habits is to pay for things by pennies. It is surprising what a number of pennies weekly wage-earners will pay if collected regularly week by week, for the purchase of things they know they will sooner or later want. It is the habit of their class, the necessity of those who possess no means for safely storing ready cash or for protecting themselves from the temptation to spend their money rashly. Thus they provide for their Christmas dinner, for their summer holiday, for their children's funerals, for their winter coals; and thus they are perfectly willing to pay for their medical attendance if we will but provide the machinery. Is it not then a trifle foolish for medical men, whilst maintaining that the working classes are able to pay medical attendance and whilst declaiming against the hospitals for treating them gratuitously, to meet together and in solemn conclave pass a resolution condemning this the only method by which payment for medical services can be obtained from the smaller weekly wage-earners?

But when the butcher sets up a penny-a-week arrangement to provide a Christmas dinner, he does not call in a committee of subscribers to manage his accounts, nor does the runner of a "goose club" ask the assistance of those who are going to eat the goose to arrange how much they shall pay for it. In each case the provider of the thing subscribed for fixes his own price, and manages the whole affair. And so must the doctors also do if they want to make this sort of thing pay. They must away with all medical aid associations and with such provident dispensaries as dispense cheap charity at the expense of their medical officers, and they must manage the whole affair themselves. Plenty of medical-aid associations are living out of the weekly subscriptions of working people, the bait for which is the medical attendance, and there is not the slightest reason why all the money so collected, except the actual cost of its collection, should not go into the pockets of the medical profession in return for medical services instead of being appropriated by the lay associations which now exploit this work. In certain towns medical men have been sufficiently energetic to take this matter of contract work into their own hands. They have organised a public medical service—a contract service—which appears to be successful in obtaining a moderate payment from a class of people who otherwise would be unable to pay at all, and in keeping the entire organisation in professional hands. What more can we want? Yet so easily are assemblies of medical men led to pass any resolution that is put before them, that the Ethical Section of the British Medical Association actually passed a resolution which struck at the root of the whole contract system including even those organisations which are worked by the medical profession and so guarded against abuse.

